# Intrahepatic T_H_17/T_Reg_ Cells in Homeostasis and Disease—It’s All About the Balance

**DOI:** 10.3389/fphar.2020.588436

**Published:** 2020-10-02

**Authors:** Hannah K. Drescher, Lea M. Bartsch, Sabine Weiskirchen, Ralf Weiskirchen

**Affiliations:** ^1^Division of Gastroenterology, Massachusetts General Hospital and Harvard Medical School, Boston, MA, United States; ^2^Institute of Molecular Pathobiochemistry, Experimental Gene Therapy and Clinical Chemistry (IFMPEGKC), University Hospital, RWTH Aachen, Aachen, Germany

**Keywords:** T_H_17 cells, T_Reg_ cells, T_H_17/T_Reg_ balance, liver, autoimmune diseases, viral infection

## Abstract

Both acute and chronic hepatic inflammation likely result from an imbalance in the T_H_1/T_H_2 cell response and can lead to liver fibrosis and end-stage liver disease. More recently, a novel CD4+ T helper cell subset was described, characterized by the production of IL-17 and IL-22. These T_H_17 cells 50were predominantly implicated in host defense against infections and in autoimmune diseases. Interestingly, studies over the last 10 years revealed that the development of T_H_17 cells favors pro-inflammatory responses in almost all tissues and there is a reciprocal relationship between T_H_17 and T_Reg_ cells. The balance between T_H_17and T_Reg_ cells is critical for immune reactions, especially in injured liver tissue and the return to immune homeostasis. The pathogenic contribution of T_H_17 and T_Reg_ cells in autoimmunity, acute infection, and chronic liver injury is diverse and varies among disease etiologies. Understanding the mechanisms underlying T_H_17 cell development, recruitment, and maintenance, along with the suppression of T_Reg_ cells, will inform the development of new therapeutic strategies in liver diseases. Active manipulation of the balance between pathogenic and regulatory processes in the liver may assist in the restoration of homeostasis, especially in hepatic inflammation.

## Introduction

CD4 T cells play a central role in mediating the host immune response to pathogens and in autoimmunity, cancer, and chronic inflammation. They maintain and enhance CD8 T cell responses, interact with B cells to induce antibody development, regulate the function of monocytes/macrophages, and orchestrate the immune response to pathogens. CD4+ T cells also modulate immune homeostasis by suppressing pro-inflammatory immune responses, build immunologic memory, and control autoimmunity (Zhu et al., 2010). These functions are achieved through the differentiation of naïve CD4+ T cells into subsets of effector, memory, and regulatory T cells.

T cell activation and differentiation rely on different stimuli, and differentiation is initiated by a cognate antigen presented by specialized antigen-presenting cells (APCs) or other immune cells. Fragmented antigens are presented on major histocompatibility complex 2 (MHC-2) molecules and recognized by the T cell receptor (TCR). Various co-stimulatory receptors and cytokines are essential for T cell activation and determine the direction of T cell differentiation.

Distinct subpopulations of CD4 T cells originating from a common precursor were first described in 1986: in mouse T cell clones, Mosmann and Coffman found that two types of T helper cells could be distinguished by their cytokine production, lymphokine activity, and transcription factor and surface marker expression (Mosmann et al., 1986). The authors defined type 1 T helper cells (T_H_1) by their secretion of interferon-γ (IFN-γ), interleukin-2 (IL-2) and tumor necrosis factor-α (TNF-α). Type 2 T helper cells (T_H_2) were characterized by the expression of IL-4, IL-5, and IL-13. Interestingly, the cytokines secreted by each mature T helper cell subset directly antagonize the development and differentiation of the corresponding opposite T helper cell subtype, thereby sustaining lineage-specific immune responses.

**Figure 1:**
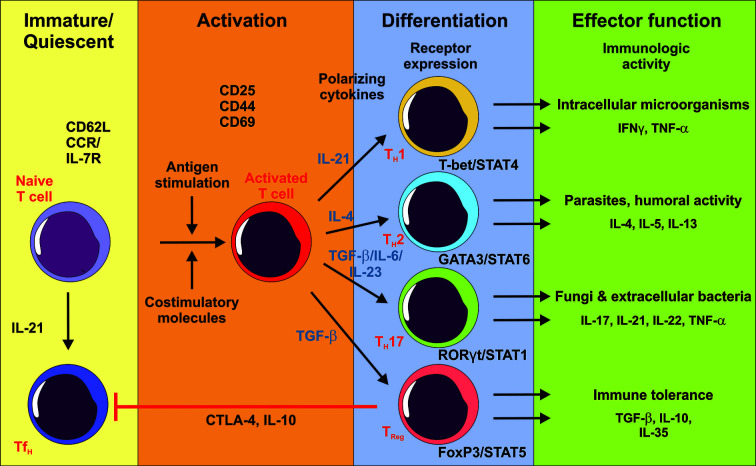
After activation of the T cell receptor by antigen stimulation and co-stimulation, immature CD4+ naïve T cells proliferate and can differentiate into different effector T cells depending on the cytokine milieu. IL-21 stimulation promotes the T_H_1 subpopulation accompanied with T-bet/STAT4 expression and effector function against intracellular pathogens. T_H_2 cell populations develop after IL-4 stimulation with GATA3/STAT6 upregulation and supporting anti-parasite immune response and humoral activity. TGF-β in the presence of pro-inflammatory cytokines promotes T_H_17 cell differentiation. T_H_17 cells support the immune response against fungi and extracellular bacteria, whereas T_Reg_ cells have an immune tolerant function by producing anti-inflammatory cytokines and inhibiting T_FH_ function. Although these specific cytokines are important for CD4+ T cell subpopulation development, the differentiation is a plastic and flexible network.

This profile has since been refined. Intensive studies of the complex cytokine milieu and transcription factor networks involved in the differentiation of CD4 T helper cell subsets originating from the same naïve CD4 T cell precursor identified many more T helper cell subsets. The potentially distinct T cell lineages include not only T_H_1, T_H_2, T_H_17, and peripheral regulatory T cells (pT_Reg_) cells, but also T_H_9, T_H_22, regulatory type 1 (Tr1), and follicular helper T cells (T_FH_) (Saravia et al., 2019). CD4+ T cell lineages are now understood to be a plastic and flexible network. One CD4+ subset cannot differentiate from only one distinct precursor cell, but rather differentiates from different subsets depending on the environmental milieu (**Figure 1**).

In this review, we discuss developmental differences between T_H_17 and T_Reg_ cells and their roles in health and disease, with a focus on liver disease. The fragile balance between these two cell types was recently found to play a crucial role in maintaining immune homeostasis. A shift of this balance drives pro-inflammatory immune responses, especially in chronic inflammatory diseases, cancer and autoimmunity. Here, we highlight the intrahepatic effects of this balance in acute and chronic inflammation as well as in liver cancer and autoimmunity.

## T_H_17 Cells in Health and Disease

A T helper cell subset of IL-17 producing T_H_17 cells defends against fungal and extracellular bacterial infection and are integral in tissue inflammation and autoimmune diseases (Tesmer et al., 2008). While it was first believed that these cells are an inflammatory subset within the T_H_1 lineage, it was later determined that T_H_17 cells are an independent T helper cell lineage (Aarvak et al., 1999).

### T_H_17 Cell Development

The discovery of a new T helper cell subset with pro-inflammatory properties, referred to as T_H_17 cells because of their expression of IL-17A, revolutionized the understanding of the adaptive immune system in the early 2000s (Park et al., 2005). T_H_17 cell differentiation in secondary lymphoid organs depends on an inflammatory cytokine milieu consisting of IL-23, TGF-β, IL-6, IL-1β, and IL-21 to activate the expression of the lineage-specific transcription factor RORγt, fostering T_H_17 cell generation. The role of RORγt was described by Ivanov et al. in 2006, who found this transcription factor to be expressed by IL-17 producing T helper cells in the lamina propria; in RORγt-deficient mice, IL-17+ cells were absent (Ivanov et al., 2006).

T_H_17 cells expand in the periphery and at the tissue site of inflammation and secrete a distinct group of effector molecules such as IL-17A, IL-17F, IL-21, IL-22, and IL-6 and express IL-23 receptor (IL-23R) on their surface (Bettelli et al., 2006; Mangan et al., 2006). Human T_H_17 cells originate from a CD161+ (the human equivalent to NK1.1) precursor in the thymus and umbilical cord blood and express CCR4 and CCR6 but not CXCR3 (Cosmi et al., 2008; Maggi et al., 2010).

Autocrine- and paracrine-derived TGF-β plays an interesting role during T_H_17 cell polarization. During the induction of RORγt expression, TGF-β synergizes with IL-6. TGF-β and IL-6 typically have opposing effects, but in this setting these proteins amplify the maturation of T_H_17 cells. In an autocrine amplification loop, TGF-β is further able to synergize with IL-21, which is predominantly produced by T_H_17 cells. This synergy promotes and enhances T_H_17 cell differentiation and pro-inflammatory immune responses (Gutcher et al., 2011). High concentrations of TGF-β in the absence of pro-inflammatory cytokines can lead to the inhibition of RORγt expression.

TGF-β can also induce the surface expression of IL-23R on differentiating T_H_17 cells, along with IL-6/IL-21, making the cells responsive to the inflammatory cytokine IL-23. IL-23 is a member of the IL-12 cytokine family and is mainly produced by APCs. It enhances the activation of STAT3, which together with RORγt stabilizes T_H_17 cell function. Therefore, IL-23 is not only important for T_H_17 cell generation and thereby the activation and maintenance of inflammatory responses at the tissue site of inflammation, but also promotes persistent chronic inflammation by supporting the proliferation of T_H_17 cells within the activated memory T cell pool (Aggarwal et al., 2003; Zhou et al., 2007; McGeachy et al., 2009).

In 2007, McGeachy et al. and Korn et al. described **“**classical**”** and **“**alternative**”** modes of T_H_17 cell activation that were further supported by a study from Ghoreschi et al. in 2010 (Korn et al., 2007; McGeachy et al., 2007; Ghoreschi et al., 2010). The different modes are driven by the availability of IL-23 and TGF-β. **“**Classical**”** T_H_17 cells, which arise from naïve CD4 T cells in the presence of TGF-β and IL-6 and the relative lack of IL-23, act through nonpathogenic expression of IL-10. Absence of expression of the TGF-β RII prevents the formation of T_H_17 cells that mediate the development of experimental autoimmune encephalomyelitis (EAE) (Veldhoen et al., 2006). The idea of a nonpathogenic T_H_17 cell subtype is further underlined by the fact that in homeostasis, T_H_17 cells are present in the intestine without detrimental effects. **“**Alternative**”** T_H_17 cells mature in the presence of IL-23 and are the pathogenic T_H_17 subtype. Overall, the development of T_H_17 cells is driven by a complex equilibrium of cytokine milieu, which influences many fine-tuning processes and a spectrum of effector functions.

The secretion of cytokines from either terminally differentiated T_H_1 (IFN-γ) or T_H_2 (IL-4) cells antagonizes the expansion of other T helper cell subtypes to sustain a lineage-specific immune response during infection. The differentiation of T_H_17 cells is negatively regulated by IFN-γ and IL-4 *via* the inhibition of IL-23 and by T_Reg_ cells *via* retinoic acid and IL-2. Opposingly, IL-17 and IL-23 hamper the development of T_H_1 cells (Harrington et al., 2005; Nakae et al., 2007). Interestingly, another IFN-γ and IL-4 independent pathway controls the development of T_H_17 cells. This process is driven by an additional member of the IL-12 family, IL-27. Like IL-12 and IL-23, IL-27 is secreted by APCs and acts independently of IFN-γR, IL-6R, and T-Bet but requires STAT1. Batten et al. and Stumhofer et al. found that a lack of IL-27 signaling lead to an increase in T_H_17 cells in autoimmune encephalomyelitis and chronic encephalitis (Batten et al., 2006; Stumhofer et al., 2006).

Unlike T_H_1 and T_H_2 cells, T_H_17 cells have an unstable cytokine memory and convey a surprising capacity of late-stage plasticity in their polarization status to adapt to a changing microenvironment. Because T_H_17 cells express low levels of IL-12R, they influence a phenotype shift after IL-12 stimulation that downregulates IL-17 and makes the cells susceptible to polarizing into a T_H_1-, but not a T_H_2-, like phenotype (Lee et al., 2009). This plasticity and the synergy between T_H_1 and T_H_17 cells is important for host defense mechanisms, as shown in a mouse model of *Mycobacterium tuberculosis* infection in which an early T_H_17 immune response recruited T_H_1 cells to the site of inflammation and promoted the development of T cell memory (Khader et al., 2007). It can also be a driving mechanism in autoimmunity and cancer.

### T_H_17 Cells in Host Defense and Autoimmunity

Until 1996, it was assumed that autoimmune diseases are the consequence of a dysregulation of T_H_1 responses. A study by Ferber et al. showed that loss of IFN-γ did not prevent the development of EAE but rather worsened disease progression (Ferber et al., 1996). Based on those findings, Oppmann and colleagues described IL-23 as new cytokine secreted by dendritic cells (DCs) that can induce the production of IFN-γ and IL-17 (Oppmann et al., 2000). Antibody-mediated blockade of IL-23 and generation of IL-23 deficient mice highlighted its involvement in the development of Crohn**’**s disease, psoriasis, EAE, and collagen-induced arthritis and the experimental animals either showed delayed and reduced disease severity or never developed autoimmune disease (Cua et al., 2003; Murphy et al., 2003; Mannon et al., 2004; Krueger et al., 2007). Clinical trials using monoclonal antibodies interfering with IL-12 and IL-23, such as ustekinumab, showed promising results in the improvement of psoriasis and psoriatic arthritis symptoms. In chronic ulcerative colitis patients, blockade of the interaction of IL-12 and IL-23 and their specific receptors on T_H_1 and T_H_17 cells also showed beneficial effects (Sands et al., 2019). Other antibodies, such as secukinumab, specifically targeting IL-17A were also highly effective in psoriasis patients (Sanford and McKeage, 2015).

Additionally, blockade of IL-17 or the loss of the regulatory mediators RORγt and IL-6 resulted in comparable outcomes ***via*** a lack of infiltration of T_H_17 cells into the tissue sites of inflammation, pointing to a crucial role for these cells in the development and progression of autoimmune diseases. Finally, experiments performing an adoptive transfer of T_H_17 cells clearly showed that these cells, but not T_H_1 cells, modulate autoimmune conditions in mice. This finding was supported in human patients with multiple sclerosis, rheumatoid arthritis, and psoriasis, in whom increased levels of IL-17 and IL-23 were observed (Ziolkowska et al., 2000; Cho et al., 2004; Vaknin-Dembinsky et al., 2006). Although an increasing body of evidence points to a dominant role of T_H_17 cells as inducers of autoimmunity, it is important to note that T_H_1 cells are also crucial in the development of autoimmunity. Each target tissue of inflammation actively participates in the formation of a site-specific cytokine milieu through its cellular composition.

The gut is an especially interesting organ to investigate the plasticity of T_H_17 cells. In homeostasis, the intestine is the primary site of T_H_17 cell differentiation, and the gut microbiota heavily influences its regulation. The TCR of intestinal T_H_17 cells has a distinct specificity for antigens coming from segmented filamentous bacteria, suggesting that these bacteria are critical for the induction of gut-resident T_H_17 maturation (Huber et al., 2012). The function of these cells under non-pathogenic conditions is to protect against microbial invasion and maintain intestinal barrier function as well as to maintain other barrier sites of the body such as the lung epithelium or the skin. Non-pathogenic characteristics of these cells can specifically be found in the small intestine, where they limit inflammation in response to bacterial or parasitic infections ***via*** the secretion of IL-10. The fragile and complexly controlled phenotype of T_H_17 cells can easily shift to an activated state in which cells attain pathogenicity and induce tissue inflammation that often leads to autoimmunity in the intestine and in other distant organs (Wu et al., 2010). In Peyer**’**s Patches, T_H_17 cells transition into a phenotype similar to T_FH_ cells producing IL-21 and Bcl-6 that can induce the production of IgA antibodies by germinal center B cells (Hirota et al., 2013). Upon bacterial infection in the colon, T_H_17 cells can transform into cells producing IL-17 and IFN-γ simultaneously in an IL-23 dependent manner and further develop into a TH1-like cell type. Transferring this cell type can lead to a T_H_17**/**T_H_1 cell-induced transfer-colitis (Morrison et al., 2013; Harbour et al., 2015).

In humans, T cells expressing IL-17 and IFN-γ were found in peripheral blood and the gut lamina propria of patients with inflammatory bowel disease (Globig et al., 2014). T_H_17**/**T_H_1 cells are present at the organ site of inflammatory responses in different models of autoimmune diseases, such as in the colon in chronic colitis or as revealed by single-cell RNA-sequencing analysis in experimental autoimmune encephalitis (EAE), which serves as model for human multiple sclerosis (Neurath et al., 2002; Gaublomme et al., 2015). Hirota and colleagues demonstrated that these cells derive from T_H_17 rather than from T_H_1 cells (Hirota et al., 2011). This was further supported by a study from Bettelli **et al**. who showed that animals deficient for the transcription factor T-bet were protected against the development of EAE (Bettelli et al., 2004).

These findings suggest that T cells expressing IL-17 and IFN-γ, also known as double producers, are highly pathogenic and predominantly involved in autoimmune diseases and tissue inflammation. However, it is not fully understood whether the production of IFN-γ by T_H_17 cells serves to limit T_H_17 cell-induced inflammation or rather promotes inflammation.

## Regulatory T Cells (T_Reg_) in Health and Disease

### T_Reg_ Development

**Figure 2:**
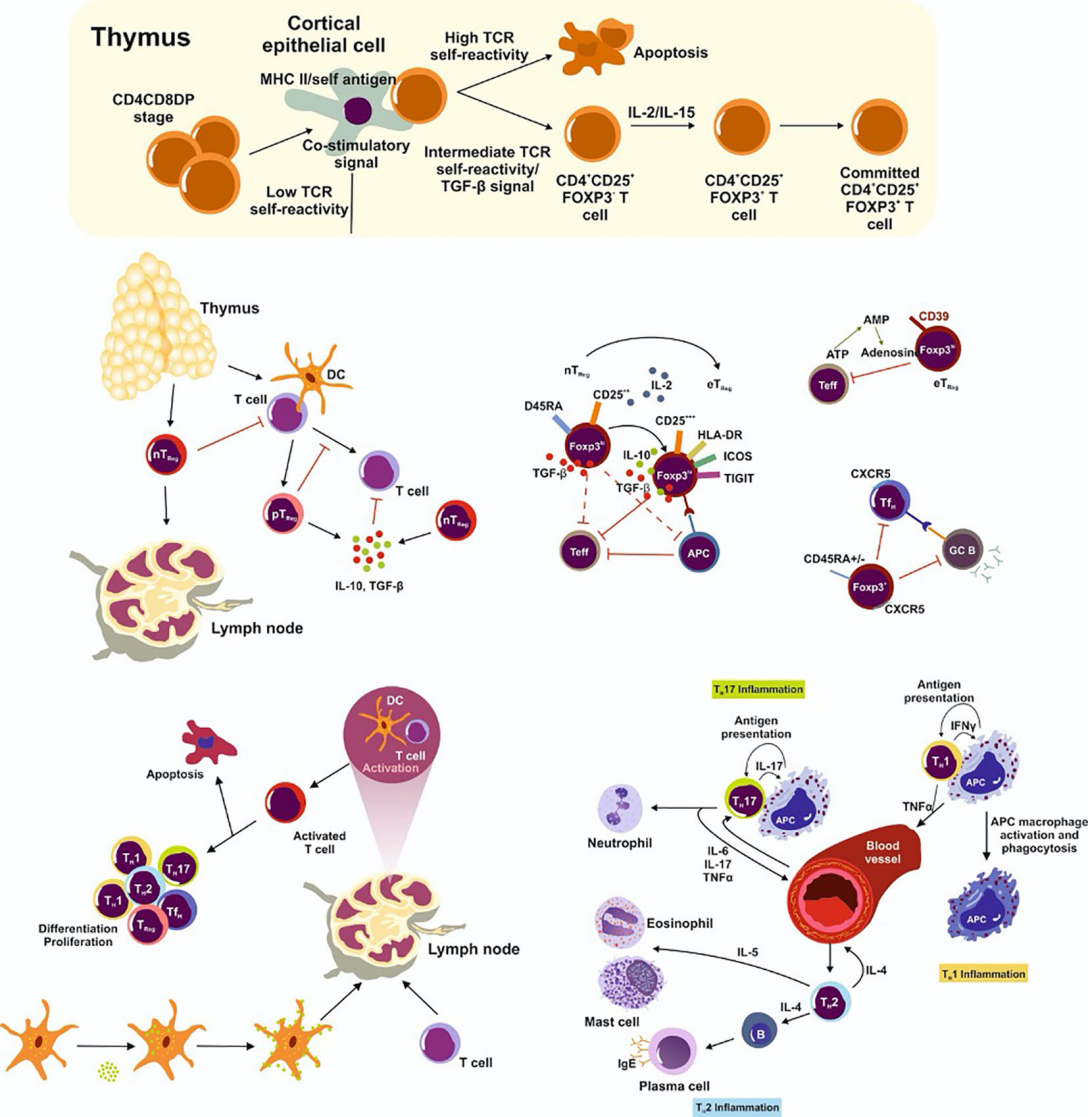
Natural T_Reg_ cells (nT_Reg_) maturate in the thymus. Their development requires the interplay of TCR-dependent recognition of self-antigen, a specific cytokine milieu (including TGF-β, IL-2, IL-7, IL-15) and the presence of co-stimulatory factors. They then infiltrate into the periphery. In the lymph node, T cells encounter a specific antigen from antigen presenting cells and become activated. Depending on the strength of the TCR binding and the presence or absence of co-stimulatory factors, cells either differentiate and proliferate into effector cells or undergo apoptosis. Peripheral T_Reg_ (pT_Reg_) cells develop predominately in the periphery from naïve CD4+ T cells. However, nT_Reg_ and pT_Reg_ cells share the lineage markers CD25 and FoxP3 which are highly expressed in effector T_Reg_ cells (eT_Reg_). eT_Reg_ cells suppress antigen presenting cells (APC) and effector T cells (T_eff_) and are thereby very efficient in inhibiting the pro-inflammatory immune response. Furthermore, T_Reg_ cells express CD39 on the cell surface to convert extracellular ATP (eATP) to adenosine and prevent a pro-inflammatory immune response. Follicular helper T (T_FH_) cells suppress the germinal center B cell reaction. Importantly, T_Regs_ also inhibit T_eff_ in the circulation. T_H_17 cells secret pro-inflammatory cytokines and attract neutrophils to the inflammation site, while T_H_1 cells promote macrophage development and function and T_H_2 cells lead to plasma, eosinophil, and mast cell differentiation and generate a pro-inflammatory immune response.

Regulatory T cells (T_Reg_) balance host defense against foreign pathogens, foster immune tolerance, and orchestrate immune homeostasis. The two main T_Reg_ subsets are natural T_Reg_ cells (nT_Reg_), which provide central tolerance against self-antigens, and peripheral T_Reg_ cells (pTReg), which develop extrathymically from conventional T cells and recognize non-self-antigens (Sakaguchi, 2004). T cells maintain peripheral tolerance by regulating inflammatory responses against the microbiota, commensals, and pathogens (Hadis et al., 2011; Lathrop et al., 2011) (**Figure 2**).

#### Natural T_Reg_ Development

nT_Regs_ develop during the neonatal period during thymocyte maturation (Liston et al., 2008). Stable nT_Reg_ development requires a complex network of antigen presenting cells (APCs) and 1) TCR-dependent recognition of self-antigens, 2) cytokine stimulation (IL-2, IL-15, and IL-7), and 3) other costimulatory signals (Bayer et al., 2005). The TCR repertoire of nT_Reg_ cells varies from the effector T cell repertoire with only minor overlap (Wong et al., 2007). The recognition affinity of self-antigens by nT_Reg_ cells is crucial for nT_Reg_ development and must fall between positive and negative selection (Maloy and Powrie, 2001). In addition to affinity to the MHC self-antigen complex, the expression level on the APC is important for optimal nT_Reg_ maturation. Cytokine stimulation by IL-2, IL-15, and IL-7 promote nT_Reg_ development, function, and homeostasis (Vang et al., 2008). Costimulatory signals through CD28 stimulation are also essential to maintain nT_Reg_ survival and to avoid defective nT_Reg_ development (Tai et al., 2005). Thus, costimulatory signals function as expansion, rather than as selective, signals.

During thymic differentiation of nT_Reg_ cells, immature single-positive CD4+ T cells express the IL-2 receptor α chain (CD25) (Setoguchi et al., 2005). CD25 has high IL-2 affinity, is continuously expressed on T_Reg_ cells, and benefits T_Reg_ responsiveness in comparison to effector cells when IL-2 concentrations are low. CD25 deficiency is related to defective T_Reg_ development, function, and an imbalanced immune system (Goudy et al., 2013). After differentiation, nT_Reg_ cells stabilize their lineage specific transcription factor forkhead box protein 3 (FOXP3) and gain suppressive functions (Fontenot et al., 2017). They further exhibit a specific CpG hypomethylation at three conserved non-coding DNA sequences (CNS) at the FOXP3 promotor, influencing the overall transcriptional activity of the cell (Toker et al., 2013). These epigenetic modifications are obligatory for T_Reg_ lineage stability, because they influence the activity of central signaling pathways like NF-κB, NFAT, STAT5, mTOR, and the binding of transcription factors to the FOXP3 promotor (Zheng et al., 2010). Further, T_Reg_-specific demethylation regions (TSDRs) contribute to a specific demethylation signature abundant in T_Reg_ function-defining genes (e.g., CTLA-4, IL-2RA, IKzf4) which regulate the overall transcriptional activity, development, and function of T_Reg_ cells.

Although FOXP3 is interrelated with T_Reg_ function, its expression is not exclusive for T_Reg_ cells as it can be transiently upregulated in activated T cells and likewise several T_Reg_ specific genes are FOXP3 independent. However, FOXP3 is necessary but not sufficient to induce T_Reg_ cells (Hill et al., 2007).

#### Peripheral T_Reg_ Development

In contrast to nT_Reg_ cells, pT_Reg_ cells develop in the peripheral tissue from naïve CD4+ T cells across the lifespan of an individual. pT_Reg_ cells also have a slightly different TCR repertoire and prevent an overwhelming immune response from occurring in response to microbiota, commensals, and pathogens (Hadis et al., 2011; Lathrop et al., 2011). pT_Reg_ and nT_Reg_ cells share the expression of the lineage defining molecules CD25 and FOXP3. Although, the frequency of pT_Reg_ cells is low, their percentage can be enriched in different tissues under inflammatory conditions (Curotto de Lafaille et al., 2008). Like nT_Reg_ cells, the promotion of pT_Reg_ differentiation is driven by TCR signaling, TGF-β, and IL-2 and costimulatory signals (Chen et al., 2003). The importance of TGF-β signaling was demonstrated in mice, when TGF-β deficiency prevented pT_Reg_ differentiation and FOXP3 stabilization (Marie et al., 2005) suggesting that specific demethylation at the FOXP3 promotor is important for pT_Reg_ differentiation, function, and lineage stability (Takimoto et al., 2010).

pT_Reg_ development can differ by tissue. The process is highly induced in the intestine because of the special immune demands at this site. Primarily, mucosal DCs support pT_Reg_ development by producing TGF-β and retinoic acid (Coombes et al., 2007). The latter supports FOXP3 stabilization by CNS1 (Mucida et al., 2009). In addition, pT_Reg_ differentiation is enhanced by metabolites produced by the microbiota in the intestine, along with chromatin structure and FOXP3 stabilization facilitated by short chain fatty acids (Arpaia et al., 2013). Fascinatingly, a high percentage of the pT_Reg_ population in the intestine co-expresses RORγt and FOXP3 while preserving the overall epigenetic and genetic signature and function of T_Reg_ cells (Yang et al., 2016).

The peripheral FOXP3+ cell population is very heterogenous and can be divided into different subpopulations according to FOXP3, CD25, and CD45RA expression profiles reflecting their activation, cytokine expression, and manifestation of epigenetic changes. CD45RA^+^FOXP3^low^CD25^low^ are defined as resting or naïve T_Reg_ cells. CD45RA^-^FOXP3^high^CD25^high^ are described as effector T_Reg_ cells (eT_Reg_), and CD45RA^-^FOXP3^low^CD25^low^ cells are non T_Reg_ cells and represent activated conventional T cells. The CD45RA^+^FOXP3^low^CD25^low^ subpopulation is further characterized by the expression of naïve T cell markers, the majority of which express CD31, a thymic emigrant marker, and the TSDR is widely conserved. The CD45RA^-^FOXP3^high^CD25^high^ subpopulation, in contrast, exhibits a highly suppressive and proliferative capacity and is profoundly demethylated (Miyara et al., 2009).

### The Role of T_Reg_ Cells in Immune Homeostasis and Inflammation

T_Reg_ cells balance the immune response in homeostasis and inflammation. These cells orchestrate the immune response of effector T cells (T_eff_) and initiate anti-inflammatory mechanisms.

#### Basic Mechanism of T_Reg_ Function

T_Reg_ cells influence the immune response by producing anti-inflammatory cytokines such as IL-10 and TGF-β. IL-10 has a potent immunosuppressive function by inhibiting the production of pro-inflammatory chemokines and cytokines and establishing immune balance in response to a pathogen, autoimmune disease, and allergy (O’Garra et al., 2004). IL-10 directly inhibits co-stimulation ***via*** CD28 and ICOS and indirectly by the downregulation of co-stimulatory molecules on APCs (Taylor et al., 2007). In addition, IL-10 produced by T_Reg_ cells orchestrates antibody production in allergies from IgE toward IgG4. IgG4 and IL-10 production is upregulated in a course of allergen-specific immunotherapies and inhibits IgE-mediated anaphylaxis (Epp et al., 2018).

Interestingly, T_Regs_ producing TGF-β may not be required for complete T_Reg_ function and influence overall T_Reg_ differentiation (Piccirillo, 2008). Numerous studies revealed the importance of TGF-β mediated T_Reg_ function and the upregulation of TGF-β to amend their suppressive function. TGF-β represses the cytolytic function of effector CD8+ cells by downregulating cytolytic genes (e.g., Fas ligand, perforin, granzyme A, B and IFN-γ) in autoimmune diseases and cancer (Thomas and Massague, 2005). In addition, TGF-β produced by T_Reg_ cells inhibits natural killer cell function and contributes to the overall anti-inflammatory effects of TGF-β (Cortez et al., 2017). Furthermore, T_Reg_-mediated TGF-β can suppress naïve T cell activation and differentiation and can function as a self-regulating stimulus to maintain T_Reg_ development (Tran, 2012). Although the role of TGF-β in direct suppressive T_Reg_ function remains controversial, this cytokine seems important but not obligatory.

Another anti-inflammatory cytokine that complements the inhibitory repertoire of T_Reg_ cells is IL-35. IL-35 plays a suppressive role in autoimmune diseases, allergies, and cancer models. In addition to T_Reg_ cells, IL-35 is secreted by B_Reg_ cells and CD8+ cells. Thus, this cytokine prevents effector T cell expansion, cytokine production, and T_H_17 differentiation (Collison et al., 2007; Niedbala et al., 2007). IL-35 also supports T_Reg_ and B_Reg_ expansion and activation by influencing the immune response in autoimmunity (Dambuza et al., 2017). In sum, cytokines fundamentally contribute to suppressive T_Reg_ function.

T_Reg_ cells express various inhibitory receptors on the cell surface. One of the most important and well-studied inhibitory receptors is the cytotoxic T lymphocyte antigen-4 (CTLA-4), which is functionally and structurally related to CD28 and can bind B7 with a 50–100-fold higher affinity. CTLA-4 is upregulated in activated and exhausted T cells but continuously expressed on T_Reg_ cells and supports their inhibitory function. By binding to B7, CTLA-4 inhibits T cell activation, proliferation, and cytokine production including IL-2 (Krummel and Allison, 1996). CTLA-4 further leads to the removal of costimulatory receptors on APCs (Sansom, 2015). A defect in CTLA-4 function, for example by non-sense mutation in the gene encoding CTLA-4, leads to defective T_Reg_ function and is accompanied by complex autoimmune disorder and immunodeficiency in humans. Interestingly, patients had a higher T_Reg_ abundancy but decreased CTLA-4 expression on the T_Reg_ cell surface. Patients with the inherited heterozygous loss of function mutation develop systemic autoimmune disorders like type 1 diabetes, autoimmune thyroid disease, systemic lupus erythematosus, and inflammatory bowel disease (Kuehn et al., 2014; Schubert et al., 2014).

T_Reg_ cells also express the inhibitory receptors programmed cell death protein 1 (PD-1) and lymphocyte-activation gene function 3 (LAG-3). PD-1 and FOXP3 work collaboratively to maintain immune tolerance, with PD-1 important to maintaining the activation balance between effector T cells and T_Reg_ cells (Zhang B. et al., 2016). In a course of anti-PD-1 therapy in cancer, PD-1+ T_Reg_ cells were amplified and mediated cancer growth (Kamada et al., 2019). LAG-3 contributes to immunosuppressive T_Reg_ function in a tumor environment and promotes maternal tolerance during pregnancy (Camisaschi et al., 2010; Zhang and Sun, 2020). Hence, LAG-3 inhibits DC maturation and function. Thus, inhibitory receptors play a fundamental role in T_Reg_ function but remain poorly understood.

In addition to cytokines and inhibitory receptors, T_Reg_ cells use metabolic disruption to influence the immune response. IL-2 is one of the most important cytokines for T cell expansion and is mandatory for T_Reg_ function and differentiation (Davidson et al., 2007). T_Reg_ cells express the high-affinity IL-2 receptor CD25 on their surface and could have a metabolic advantage in comparison to effector T cells, especially in a milieu where the IL-2 concentration is low. Accordingly, cytokine deprivation by T_Reg_ cells induced apoptosis in effector T cells (Pandiyan et al., 2007). In contrast, IL-2 consumption was not required for T_Reg_ suppression (Oberle et al., 2007). Nevertheless, modern low-dose IL-2 therapies in various diseases could demonstrate a preferential T_Reg_ expansion and thereby have a positive effect on patient outcome (Hartemann et al., 2013; Matsuoka et al., 2013; He et al., 2016).

T_Reg_ cells use the membrane-bound ectonucleotidases CD73 and CD39 to generate adenosine from extracellular ATP to influence the immune response. Extracellular ATP usually promotes inflammation, whereas adenosine leads to anti-inflammatory effects. CD39 is abundant on T_Reg_ cells, whereas CD73 is intracellularly enriched in human T_Reg_ cells and upregulated after T_Reg_ activation (Schuler et al., 2014). T_Reg_ cells are sensitive to extracellular ATP, and the upregulation of CD39 is accompanied by remission of inflammatory bowel disease (Gibson et al., 2015). Thus, CD39 signaling is primarily a mechanism to suppress T_H_17 function and development. Adenosine leads to CTLA-4 and PD-1 upregulation in T_Reg_ cells and promotes T_Reg_ suppression of DC function (Ring et al., 2015).

The first indication that cytolytic mechanisms play a role in T_Reg_ function came from studies of granzyme B. In particular, granzyme B is upregulated in activated T_Reg_ cells and mediates suppression of B cell function, and granzyme B deficiency reduces T_Reg_ suppression. Granzyme B-expressing T_Reg_ cells are enriched in human colorectal cancer and potent suppressors of effector T cells. Further, T_Reg_ cells protect themselves from granzyme B-mediated killing by upregulating serine protease inhibitor 6 (Azzi et al., 2013; Sun et al., 2020).

#### T_Reg_ Function in Autoimmunity

Defects in molecules important for T_Reg_ function can lead to autoimmune diseases, underlying the importance of T_Reg_ effector proteins in pathophysiology. For example, the inherited IPEX syndrome (X-linked autoimmune syndrome immunodysregulation polyendocrinopathy enteropathy X-linked) is caused by different loss of function mutations in the FOXP3 gene. These defects can further lead to the development of autoimmune diseases like diabetes type 1, autoimmune colitis, or hepatitis (Le Bras and Geha, 2006). IL-2RA mutations cause a phenotype similar to IPEX syndrome and CD25 deficiency can increase the vulnerability to viral infection (Goudy et al., 2013). In addition to these monogenetic T_Reg_ diseases, T_Reg_ dysfunction appears in other immune deficient syndromes. Autoimmune diseases like type 1 diabetes, multiple sclerosis, systemic lupus erythematosus, myasthenia gravis, and rheumatoid arthritis are associated with altered T_Reg_ quantity or quality (Wakabayashi et al., 2006; Lapierre et al., 2013).

The direction of T_Reg_ alteration is controversial. Different studies show either an increase or decrease in T_Reg_ cell numbers based on the stage of the disease and the heterogeneous use of T_Reg_-defining molecules. In addition, as explained above, T_Reg_ defining molecules can be upregulated after general T cell activation in humans (Pillai et al., 2007). Nevertheless, T_Reg_ cells are an important target for therapy of autoimmune diseases and remission can partly reverse the defective T_Reg_ function (Hartemann et al., 2013; Humrich et al., 2015; Rosenzwajg et al., 2015; He et al., 2016). Treatment with tocilizumab in patients with rheumatoid arthritis, for example, increased T_Reg_ frequency and restored T_Reg_ function (Kikuchi et al., 2015). Another therapeutic approach is the adoptive transfer of ***in vitro***-expanded T_Reg_ cells. This strategy was beneficial in mouse models and is now being tested in humans (Morgan et al., 2005; Mathew et al., 2018).

Tumor tissue is especially challenging for the immune system. T_Reg_ cells impair the immune response against tumor antigens by effector T cells that evolve from potential self-reactive cells. In general, a T_Reg_-enriched tumor tissue with decreased abundancy of CD8+ cells is associated with poor prognosis, metastasis, and reduced survival (Mougiakakos et al., 2010). Tumor-infiltrating T_Reg_ cells are primarily effector T_Reg_ cells (CD45RA-FOXP3highCD25high), which are active and proliferative. Furthermore, these cells differ in the expression of activation markers, T_Reg_ markers, and inhibitory receptors on their cell surface. Tumor-infiltrating T_Reg_ cells also express several chemokine receptors such as CCR4 and CCR8 (De Simone et al., 2016). In the tumor, T_Reg_ cells interact with immune cells, cancer cells, and fibroblasts to influence tumor immunity. Tumor-associated fibroblasts promote tumor progression and positively regulate T_Reg_ function and frequency (Kato et al., 2018). Furthermore, T_Reg_ cells and cancer cells bidirectionally support their growth and function. Immunosuppressive T_Reg_ function enables tumor progression, while cancer cells secrete TGF-β, IDO, and COX-2 to promote T_Reg_ trafficking and differentiation (Costa et al., 2018). Adenosine is produced by cancer cells and T_Reg_ cells and functions as a mediator, encouraging cancer cell growth, angiogenesis, and metastasis (Chimote et al., 2018). In addition to cancer cell effects, T_Reg_ cells have a tremendous impact on tumor-infiltrating immune cells. They reciprocally promote nonclassical monocytes, B_Reg_ differentiation, and MDSCs trafficking to create an immunosuppressive cell composition. Furthermore, T_Reg_ cells inhibit proinflammatory immune cells such as NK cells and cytotoxic lymphocytes to prevent an effective anti-tumor immune response (Chang et al., 2016; Sarhan et al., 2018). Modern immunotherapies use anti-CTLA-4 and anti-PD-1, which mainly target the T_Reg_ immune response (Ha et al., 2019).

An important cell subpopulation that balances the immune response in the course of pathogen infection and vaccination are follicular regulatory T cells (T_FR_). T_FR_ cells affect the humoral immune response by influencing B cell maturation in the germinal center (GC). During this process, B cells undergo somatic hypermutation to establish a high affinity and effective humoral immune response. This development is supported by T_FH_ cells and is tightly regulated by T_FR_ to prevent autoimmunity and an imbalanced immune response (Wollenberg et al., 2011). T_FR_ influence T_FH_ frequency and function and in addition B cells directly by inhibiting their activation. Interestingly, T_FR_ express a distinct TCR repertoire for foreign antigens and potential self-antigens. T_FR_ resemble T_FH_ cells in CXCR5, ICOS, and BCL-6 expression but T_FR_ also express CD28, FOXP3, and Blimp1 (Chung et al., 2011). T_FR_ cells differentiate from CD25+FOXP3+ cells and function as an effector T_Reg_ subpopulation (Linterman et al., 2011). CTLA-4 is the most important mode of T_FR_-mediated immune cell regulation, but PD-1 also contributes to full T_FR_ function (Sage et al., 2014). T_FR_ and T_FH_ influence one another reciprocally, and the interaction can be influenced by the microenvironment. In a recently published study, different adjuvants balanced T_FR_ frequency and function during vaccination (Bartsch et al., 2020). The T_FR_ function was affected by IL-6 signaling and the altered T_FR_ cell frequency influenced antibody glycosylation and the overall humoral immune response (Bartsch et al., 2020).

In addition to the GC reaction, T_FR_ can be detected in the blood even at low frequency. Circulating T_FR_ cells have a memory-like phenotype and fine tune the secondary response to an antigen by influencing reactivation of DCs to GC and cytokine production, antibody class-switching, and B cell activation (Sage et al., 2014). T_FR_ dysfunction can be associated with autoimmune diseases, graft versus host reactions, and allergies (He et al., 2013). Overall, T_Reg_ cells play an important role in orchestrating the immune response in health and disease.

## The T_H_17/T_Reg_ Cell Balance in the Liver

The liver is an immunogenetic organ exposed to a variety of antigens and pathogens from the digestive tract and is essential to building an effective immune response. Interestingly, and in contrast to the blood, the CD4/CD8 ratio is reversed in the liver (Doherty and O’Farrelly, 2000). The liver is naturally enriched with innate immune cells, namely, macrophages (Kupffer cells), natural killer (NK) cells, and NK T cells. Especially during fibrogenesis, infiltrating monocytes are important for continuous inflammation and the activation of extracellular matrix producing hepatic stellate cells (HSCs). The adaptive immune system also plays a critical role in these processes. The intrahepatic T_Reg_ frequency can differ from 1% to 5**%** among all intrahepatic lymphocytes (Oo et al., 2012). The expression of IL-17RA was observed on parenchymal and non-parenchymal cells including hepatocytes, HSCs, Kupffer cells, and endothelial cells, all of which exacerbate inflammatory reactions upon injury. In two different mouse models of hepatic fibrosis (bile duct ligation and carbon tetrachloride), the deletion of IL-17A, IL-23, and IL-17RA inhibited HSC activation and fibrosis development. This finding implies a direct functional link between IL-17A mediated stimulation of HSCs by activation of STAT3-dependent signaling (Meng et al., 2012). Further, isolation experiments in primary liver-resident cells revealed IL-17 production and IL-17 signal transmission by almost all liver-resident cell types. Kupffer cells especially express high levels of IL-17 and show significant upregulation of IL-17 and IL-1β upon stimulation. mRNA expression of IL-17RA and IL-17RC could additionally be found in hepatocytes, Kupffer cells, HSCs, and liver endothelial cells. Despite IL-17RA expression, hepatocytes and liver endothelial cells do not express IL-17 themselves (Zenewicz et al., 2007). Another study using a cell transplantation model pointed to a direct interaction between HSCs and T_Reg_ cells. Jiang et al. found that upon transplantation, HSCs in allogeneic recipients convey the selective expansion of CD4^+^CD25^+^FoxP3^+^ cells in an IL-2-dependent fashion to protect parenchymal cells from rejection (Jiang et al., 2008).

In contrast, CD4+ and CD8+ T cells, NK T cells, γδT cells, neutrophils, and macrophages do express IL-17 in the liver. The equilibrium of T_H_17 and T_Reg_ cells is regulated not only by differentiation but also at the epigenetic level. Interestingly, recent studies show that in the presence of IL-1β, IL-2, IL-21, and IL-23, IL-17 producing cells can also develop from T_Reg_ cells due to a differentiation switch that removes their suppressive function (Koenen et al., 2008; Deknuydt et al., 2009).

**Figure 3:**
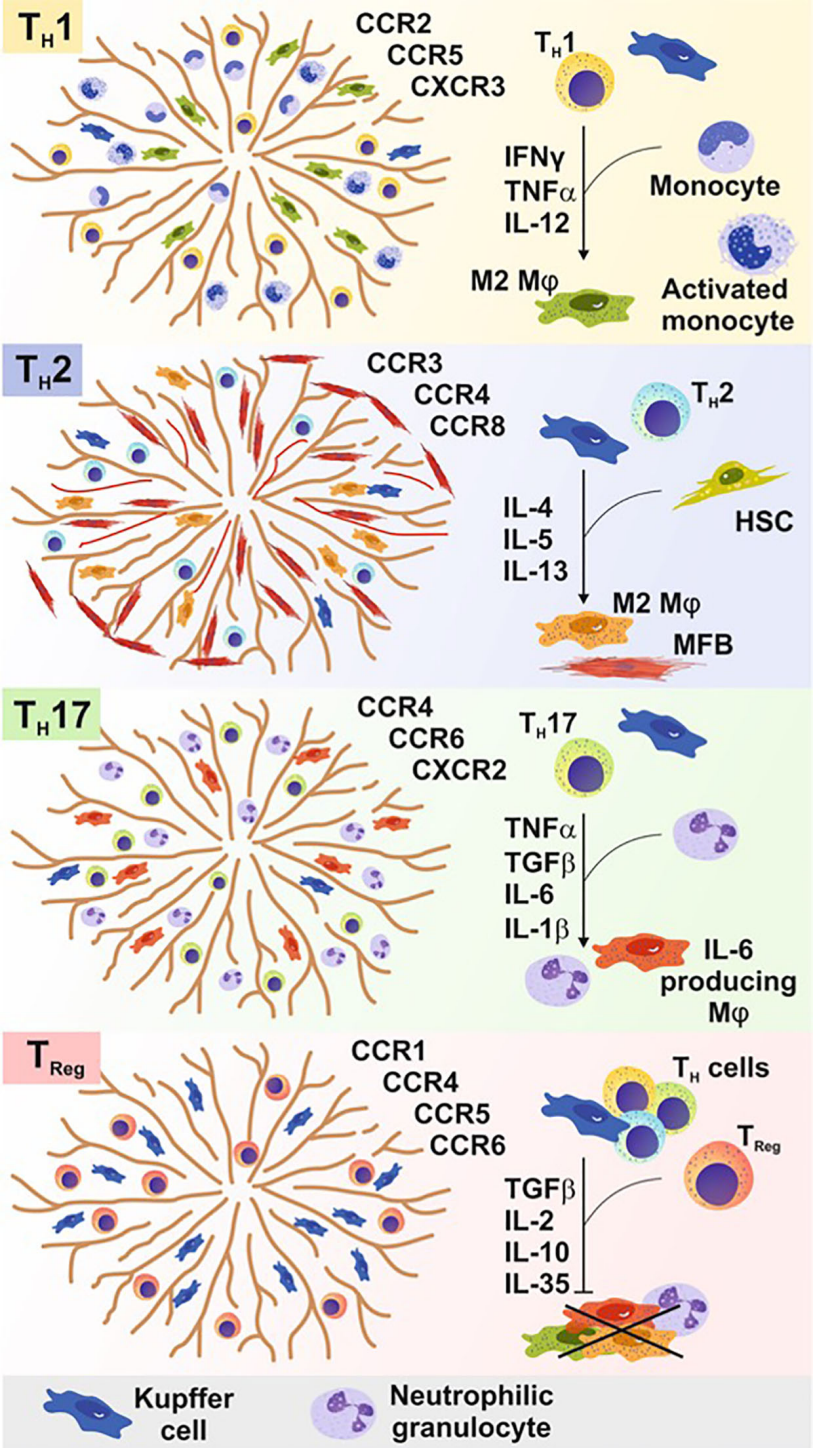
In acute and chronic liver diseases, T cells are crucial to either initiate, maintain, or terminate pro-inflammatory immune responses. Different T cell subpopulations develop, expand, and function based on a distinct cytokine milieu influenced by the presence of different types of immune cells. Different CD4+ cells recognize certain chemokine receptors within the liver and are thereby attracted by different cell types and stimuli to infiltrate the liver tissue. The differentiation of these subsets requires a specific cytokine milieu generated by non-parenchymal cells (e.g. monocytes) or parenchymal cells [e.g., hepatic stellate cells (HSCs)]. T_H_1 cell cells promote alternatively activated macrophage development (M2), whereas T_H_2 cells additionally promote liver myofibroblast (MFB) activation. T_H_17 cells leads to the recruitment of neutrophils, granulocytes, and macrophages to the site of inflammation to induce and sustain a pro-inflammatory immune response. Regulatory T (T_Reg_) cells and follicular T (T_FH_) cells prevent the pro-inflammatory immune response of several parenchymal and non-parenchymal cells within the liver tissue in course of an inflammatory immune response.

T_Reg_ and T_H_17 cells are also often significantly increased in chronic inflammatory liver diseases and are important to balance the persisting pro-inflammatory immune response. Interestingly, a reciprocal relationship between T_H_17 and T_Reg_ cells exists in their differentiation as in their effector function (**Figure 3**).

T_Reg_ and T_H17_ cells can co-express the lineage defining transcription factors RORγt and FOXP3. Environmental conditions such as the tissue-specific cytokine milieu at the site of infection can influence this expression and influence the balance by fostering either T_Reg_ or T_H_17 cell development by simultaneous inhibition of the other cell type. TGF-β, for example, is required for the differentiation of both subsets; the absence or presence of proinflammatory cytokines defines whether a T_H_17 or T_Reg_ cell develops (Yang et al., 2008; Hammerich et al., 2011). In contrast, T_H_17 cells express IL-10 receptor α, which can convey a T_Reg_-induced decrease in T_H_17 cells in an IL-10-dependent manner (Huber et al., 2011). Recent studies point to a delicate balance between T_H_17 and T_Reg_ cells crucial to maintaining tissue homeostasis. In addition to IL-6, other factors such as retinoic acid, rapamycin, or cytokines (e.g., IL-2 and IL-27) influence this balance significantly. T_Reg_ cells are assigned a decisive role in hepatic immunity. Results obtained in mouse models of acute and chronic liver disease also point to a major involvement of T_H_17 and T_Reg_ cells in a variety of human inflammatory liver diseases. For example, in a cancer milieu, glucose consumption by tumor cells preferentially promotes T_Reg_ differentiation and decreases T_H_17 cell development thereby supporting the immune escape strategy of tumor cells. Another important ubiquitous environmental condition during chronic and acute infection is hypoxia. Hypoxia can induce the hypoxia inducible factor 1α (HIF-1α) as an adaptive mechanism of the cells to low oxygen concentration. HIF-1α stabilization supports RORγt and IL-17 production while targeting FOXP3 to proteasomal degradation. Manipulation of the balance between pathogenic and regulatory processes in the liver are believed to allow the focused restoration of homeostasis especially during hepatic inflammation.

The detailed analysis of T_H_17 cells in human liver remains difficult because the cell frequency is low and cells can only be analyzed after their ***in vitro*** activation with phorbol 12-myristate 13-acetate (PMA) and ionomycin. This ***in vitro*** activation is interesting but must be critically considered because the extent to which it reflects the ***in vivo*** situation and cell status upon isolation is unknown.

### Autoimmune Diseases

One example of specific immune change in the liver is autoimmune hepatitis (AIH). Although the cause of AIH is not fully understood, T cell mediated liver tissue destruction is involved and AIH could be associated with genetic and environmental alterations. AIH leads to chronic liver inflammation, circulating autoantibodies, and elevated liver enzymes (Tait et al., 1989). Zhao et al. showed that patients with AIH have increased serum levels of IL-17 and IL-23 together with an increased frequency of T_H_17 cells in the liver compared to controls. Furthermore, the frequency and function of T_Reg_ cells in the blood was decreased (Ferri et al., 2010). By analyzing the T cell composition in the liver, it was demonstrated that the total T_Reg_ number was not altered in AIH patients. In contrast, these patients displayed higher hepatic expression of the T_H_17-related cytokines IL-17, IL-23, IL-6, and RORγt. *In vitro* experiments showed that IL-17 induces IL-6 *via* MAPK signaling in hepatocytes, which in turn stimulates T_H_17 cell differentiation and infiltration in a positive feedback loop (Zhao et al., 2011). These results are supported by a retrospective study of 100 AIH patients. In addition to elevated serum levels of IL-17, IL-6, IL-21, and TNF-α, an increased frequency of T_H_17 cells was observed. Pro-inflammatory cytokines were positively correlated with liver injury, whereas IL-10 was negatively regulated with autoantibodies (An, 2019). Likewise, T_Reg_ cells from AIH patients had decreased CD39 expression and functionally failed to prevent T_H_17 accumulation mediated by extracellular ATP (Grant et al., 2014). Remission in AIH patients was associated with restored T cell balance, and the infusion of ex vivo expanded T_Reg_ cells was beneficial in a murine model (Lapierre et al., 2013). In sum, the balance of T_Reg_ and T_H_17 composition at the site of inflammation and T_Reg_ function is critical in AIH pathomechanisms.

Primary biliary cirrhosis (PBC) is a chronic cholestatic liver disease characterized by the loss of immune self-tolerance leading to the chronic injury of biliary epithelial cells. Ninety percent of affected patients are women older than 40 years. The importance of the T_H_17/T_Reg_ balance in disease progression of primary biliary cirrhosis (PBC) is evident when considering that a knockout for CD25 (IL-2Rα) in mice serves as an animal model for this disease. Mice spontaneously develop autoantibodies caused by a loss of function of T_Reg_ cells and acquire biliary duct damage similar to that observed in PBC patients (Wakabayashi et al., 2006). Deficiency of functional T_Reg_ cells leads to elevated T_H_17 cell numbers in the liver and elevated IL-17 levels in these mice compared to wildtype controls. A possible explanation might be the missing repressive function of IL-2 during T_H_17 cell differentiation. In line with the results obtained in mice, patients suffering from liver fibrosis due to PBC show a higher frequency of T_H_17 cells in blood than healthy control patients. Liver biopsy samples of PBC patients point to a dislocation of these cells around the portal tracts (Shi et al., 2015). Patients with cirrhosis secondary to PBC displayed an even higher infiltration of T_H_17 cells into liver tissue (Tan et al., 2013). However, the exact mechanisms that cause an induction of T_H_17 cells in livers of IL-2RA knockout animals remain elusive.

**Figure 4:**
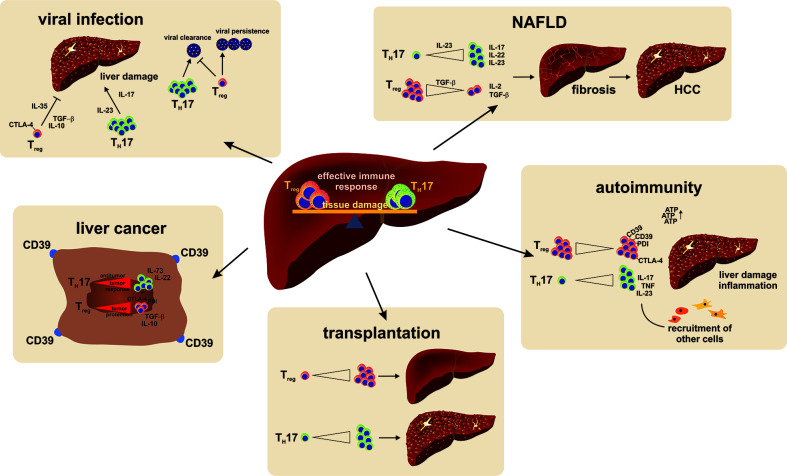
A reciprocal relationship exists between T_H_17 and T_Reg_ cells. Their balance is found to be important in the persistence or recovery from liver injury. A shift of the balance to a more dominant T_H_17 cell response favors pro-inflammatory reactions and persisting damage. In viral infections, T_H_17 are especially important for viral clearance but at the same time cause liver damage. T_Reg_ cell on the other hand prevent liver damage but also trigger viral persistence by strengthening the anti-inflammatory immune response. An imbalance of the T_H_17/T_Reg_ milieu to a dominant T_H_17 cell response favors disease development and progression in NAFLD. In autoimmune diseases a T_Reg_ cell responses have beneficial effects in regards to self-tolerance, restore immune homeostasis and are even proposed as a treatment option in acute and chronic transplant rejection reactions. In a tumor environment T_Reg_ cells support tumor cells from being targeted by the immune system and hence promote tumor growth and metastasis.

Primary sclerosing cholangitis (PSC) is another chronic-inflammatory liver disease with an unknown pathogenesis. Similar to PBC, PSC can lead to liver fibrosis and obliteration of intra-and extrahepatic bile ducts. PSC is often associated with chronic ulcerative colitis, and there is no effective treatment (Hirschfield et al., 2013). Patients with also have a decreased peripheral T_Reg_ frequency with epigenetic changes. Furthermore, a decrease in T_Reg_ numbers was associated with an IL-2RA gene polymorphism and lead to reduced T_Reg_ function (Sebode et al., 2014), (**Figure 4**).

### Acute Liver Injury

Mouse models of acute liver injury were used to further investigate the role of T_H_17 cells in the liver. In the concanavalin A- (ConA-) model of acute T cell induced hepatitis, IL-1- deficient animals were challenged and knockout mice developed less severe injury with higher T_Reg_ numbers compared to wildtype mice (Nagata et al., 2008). However, these results are controversial; another study found in the same model that IL-17-deficient mice seemed to develop a comparable level of liver injury after ConA-treatment (Zenewicz et al., 2007). In another interesting mouse model of drug-induced liver injury (halothane injection intraperitoneally), mice had increased serum levels of IL-17. After the administration of an IL-17 neutralizing antibody serum, liver enzymes AST and ALT had significantly decreased levels, with a downregulation of inflammatory cytokines such as TNF-α. These beneficial effects could be directly reversed by the application of recombinant IL-17 (Kobayashi et al., 2009).

### Viral Infection

The T_Reg_/T_H_17 balance is essential for an effective immune response and at the same time preventing excessive liver injury during viral infection. Thus, the liver is affected by several viruses, and some of them lead to persistent infection and can cause liver cirrhosis, organ failure, and cancer. During acute hepatitis A virus (HAV) infection, serum IL-17 levels are correlated with liver injury, and liver resident and circulating T_Reg_ frequencies are negatively linked to elevation of ALT and AST (Choi et al., 2015).

The major problem in persistent viral infection is a failure of the T cell response involving T cell exhaustion due to persistent antigen presentation. T_Reg_ cells also play a role in the immunopathology of persistent viral infection. It was demonstrated that liver sinusoidal endothelial cells (LSEC) are potent enough to promote T_Reg_ differentiation by the continuous induction of FOXP3 in conventional T cells, although all liver cells were able to induce T_Reg_ differentiation. T_Reg_ stabilization did not require inflammation but did require TGF-β, which is abundant on the LSEC cell membrane. Experimentally, LSEC-induced T_Reg_ cells expressed FOXP3 and had efficient inhibitory functions on effector T cells *in vitro* and *in vivo* (Carambia et al., 2014). Antigen presentation on LSECs and thereby an early T_Reg_ development in a course of sub-infectious viral infection of hepatotropic viruses, e.g., chronic hepatitis C virus (HCV), can support ineffective virus clearance and chronic infection (Park et al., 2013). In contrast, virus-specific T_H_17 cells were correlated with liver injury and inflammation, but T_H_17 quantity could not be linked to effective viral clearance. Furthermore, IL-23 and IL-17 levels in HCV-infected patients were elevated, and IL-23 therapy was reported to modulate the antiviral response by preferentially promoting T_H_17 immune cells (Meng et al., 2016). In a course of HCV infection, T_Reg_ cells were mainly found in necro-inflammatory liver areas to inhibit the effector CD8 T cell response, which is the main cause of liver damage in HCV and hepatitis B virus (HBV) infection. Isolated T_Reg_ cells from HCV-infected patients suppressed virus-specific CD8+ cells, whereas the depletion of T_Reg_ cells increased their proliferation. Furthermore, TCR analysis demonstrated that the effective T_Reg_ population during chronic HCV infection is heterogeneous and consists of nT_Reg_ and pT_Reg_ cells (Losikoff et al., 2012). In comparing the T_Reg_ cells of patients who spontaneously resolved the infection and patients with persistent infection, core virus-specific T_Reg_ cells were primarily found in patients with chronic HCV infection. They inhibit the virus-specific T cell response by producing IL-10 and IL-35 (Langhans et al., 2010). Furthermore, several studies investigated a positive correlation between T_Reg_ quantity and function with chronic HCV progression (Cabrera et al., 2004; Ebinuma et al., 2008). T_H_17 cells on the other hand are enriched in livers of patients with chronic HCV infection mainly driven by Tim-3, which leads to a differential regulation of IL-12 and IL-23 (Wang et al., 2013).

The T_Reg_/T_H_17 balance also plays an important role in chronic HBV infection. T_Reg_ cells inhibit the antiviral response of effector T cells (Yan et al., 2014). The frequency of circulating T_Reg_ cells is differently described in chronic HBV infection, but there are some indications that the T_Reg_ frequency is increased in severe chronic HBV infection. Furthermore, T_Reg_ frequencies could be positively correlated with viral load, HBeAg (Hepatitis B envelope Antigen), and HBsAg (Hepatitis B surface Antigen) (Manigold and Racanelli, 2007; TrehanPati et al., 2011). Patients with a predominant T_H_17 response have high plasma viral loads. Especially in HBV infection and HBV-induced cirrhosis, IL-17+ cells increase with cirrhosis stage and the HBcAg (Hepatitis B core Antigen) mediates T_H_17 cell responses by an IL-17R-induced activation of monocytes/macrophages. This effect leads to the production of elevated levels of pro-inflammatory cytokines such as IL-6, TNF-α, IL-12, and IL-23 (Sun et al., 2012). Further, the elevation of T_Reg_ and T_FR_ cells in chronically HBV infected patients associated with elevated IL-10 and TGF-β levels in comparison to healthy individuals (Liu et al., 2020). T_Reg_ differentiation was thereby promoted by TGF-β production of hepatic stellate cells and activation of Notch signaling during chronic inflammation. In a course of antiviral response, T_H_17 cells increased in quantity accompanied with an increase in viral load (Ichikawa et al., 2011; Yan et al., 2014). In addition, in course of antiviral therapy, PD-1 expression decreased on T_H_17 cells and other effector T cells, indicating an improved T cell exhaustion; however, PD-1 was not present on T_Reg_ cells (Wei et al., 2013). The T_H_17/T_Reg_ balance is critical in the development of liver cirrhosis in chronically-infected patients. An imbalance in T_H_17/T_Reg_ cells was thereby an independent predictive factor for decompensated liver cirrhosis (Lan et al., 2019). In addition, Yang et al. demonstrated that IL-35 is responsible to balance the T_Reg_ and T_H_17 balance in acute and chronic HBV infection by preferentially increasing virus-specific T_Reg_ cells and the prevention of T_H_17 cell differentiation (Yang et al., 2019). In conclusion, T_Reg_ and T_H_17 cells contribute to the immune response during viral liver infection and the optimal balance is important for an effective antiviral immune response and prevention of complications (**Figure 4**).

### Alcoholic and Nonalcoholic Steatohepatitis (ASH and NASH)

Patients with alcoholic steatohepatitis (ASH) show a direct correlation between the severity of inflammation and the amount of liver damage. The degradation of ethanol mediated by cytochrome P450 2E1 (CYP2E1) is associated with various inflammatory responses within the liver. The immune response leads to the production of reactive oxygen species (ROS) and TNF-α and the infiltration of immune cells. T cells infiltrating into the liver secrete high levels of pro-inflammatory cytokines, thereby attracting neutrophils to the tissue site of inflammation. Neutrophil recruitment could be closely linked to the prominence of T_H_17 cells. This in turn leads to increased IL-17 serum levels in ASH patients, which could be directly linked to progressive liver damage. Further, the number of T_Reg_ cells decreases in the blood of these patients (Lemmers et al., 2009).

Nonalcoholic steatohepatitis (NASH) is related to metabolic syndrome. This condition has become the most common cause of chronic liver disease and will likely be the main cause for liver transplantations within the next decade (Younossi et al., 2019). Patients with non-alcoholic fatty liver disease (NAFLD) have a high risk to progress from simple steatosis to more advanced disease stages such as NASH, cirrhosis, and hepatocellular cancer (HCC). The exact mechanisms of the pathogenesis of NASH are poorly understood. However, the role of the activation of the adaptive immune system *via* a T_H_17-mediated immune responses is becoming evident.

Targeting the balance between T_H_17 and T_Reg_ cells is a relatively new approach and currently topic of many research studies. The lack of available human tissue samples makes it difficult to investigate this as a potential new treatment option. Nevertheless, recent studies in mice and few data from humans indicate a decisive role of CD4+ T cells in the progression from NAFLD to NASH up to HCC development (Hammerich et al., 2011; Rau et al., 2016). A key mechanism within this process is a strong infiltration of neutrophils together with an increased IL-6 signaling and T_H_17 accumulation (Hubscher, 2006). This further leads to a depolarization of the intrahepatic CD4+ cell response to a more T_H_17 cell-driven reaction; at the same time, T_Reg_ cell activity is suppressed (Gomes et al., 2016; Rau et al., 2016). Although the total T_Reg_ number of circulating and intrahepatic T_Reg_ cells is not altered, the overall T_H_17/T_Reg_ balance is shifted to a more dominant pro-inflammatory immune response.

The majority of data on T_H_17 cells in NASH is limited to mice. Different mouse models for diet-induced nonalcoholic steatohepatitis, such as the methionine-choline deficient-diet (MCD-diet) and the widely used high fat diet (HFD) model, can lead to steatohepatitis and subsequent fibrosis. Disease development and progression in these models is accompanied with an increase in T_Reg_ cell numbers at early disease stages in which only steatosis is present, and shifts significantly to a more dominant T_H_17 cell-driven response at later time points when steatohepatitis and beginning fibrosis are present. In the liver fibrosis CCL4 mouse model, increased IL-17 levels led to an elevated collagen1α1 expression in HSCs triggered by STAT3 signaling (Meng et al., 2012). Gomes et al. showed in 2016 that the excess of nutrients leads to the expression of the factor unconventional prefoldin RPB5 interactor (URI) in liver of mice treated with different steatohepatitis-inducing diets. URI promoted HCC development *via* a shift of the CD4+ T cell composition during NASH and NASH-HCC development. The overexpression of human URI in mouse hepatocytes led to spontaneous development of steatohepatitis, which could be strengthened by feeding steatohepatitis-inducing diets (CD-HFD) or MCD. Disease was accompanied by increased IL-17 and T_H_17 cells in blood and liver. Mice with a heterozygous, hepatocyte-specific deficiency for URI were protected from the development of steatohepatitis together with decreased numbers of T_H_17 cells. Inhibiting the differentiation of T_H_17 cells through the blockade of RORγt lead to an improved in lipid metabolism, insulin resistance, and HCC development. The application of recombinant IL-17 in wildtype mice induced steatohepatitis, the infiltration of neutrophils to white adipose tissue, and led to an increased number of T_H_17 cells.

Interestingly, data from human NASH patients correlated with the expression of URI with high IL-17 levels and hepatic steatosis (Gomes et al., 2016). Another study points to overall diminished CD4+ T cell numbers in NASH, and that this reduction is an essential factor in the progression from NASH to HCC development (Ma et al., 2016). The observed changes in the T helper cell profile during NASH development can be caused either by a depolarization of CD4+ T cells to a T_H_17 cell phenotype, or by a relative shift of the CD4+ T cell composition in the liver due to depletion of other T helper cell subsets, or driven by an altered infiltration of distinct CD4+ T cell subsets.

A study from Rau et al. in 2016 showed that the progression from NAFLD to NASH is directly correlated with an increased frequency in T_H_17 cells in blood and liver of NAFLD and NASH patients together with an altered T_H_17/T_Reg_ balance depicted by an increased T_H_17/T_Reg_ cell ratio in both compartments (Rau et al., 2016). In visceral adipose tissue and subcutaneous adipose tissue of morbid obese patients, an increase in the mRNA expression of IL-17 was found compared to normal weight patients. The same patients also showed increased numbers of T_H_17 cells in both adipose tissues and peripheral blood mononuclear cells (PBMCs), while T_Reg_ cell numbers were decreased due to impaired survival of these cells (McLaughlin et al., 2014). A recent study published in April 2020 further points to the direct relationship between liver and adipose tissue in regulating the T_H_17/T_Reg_ balance. Van Herck and colleagues demonstrated that mice fed a high-fat high-fructose diet displayed an increase in T_H_17 cells in both compartments, with a simultaneous decrease in T_Reg_ cells. After removing the steatohepatitis-inducing diet, the disruption in the T_H_17/T_Reg_ balance persisted. The administration of an IL-17 neutralizing antibody subsequently decreased the pro-inflammatory immune response in the liver (Van Herck et al., 2020). NASH is further closely related to the development of HCC. The involvement of TH17 cells in tumor formation and patient survival was recently described as influencing the prevention of apoptosis in tumor cells induced by T_H_17 cells due to IL-17 promoting angiogenesis and an IL-23 driven tumor growth (Langowski et al., 2006; Zhang et al., 2009). Understanding the role of T_H_17/T_Reg_ balance and targeting it therapeutically is an interesting approach for the treatment of NASH (**Figure 4**).

### Liver Fibrosis and Hepatocellular Carcinoma

Chronic liver disease changes and impairs organ structure and function. Liver disease leads to tissue replacement and scaring subsequently leading to liver fibroses and cirrhosis. Several studies describe the importance of T_H_17 and T_Reg_ cells in this process. Although the exact role of T_Reg_ cells in liver fibrogenesis is not fully understood, T_Reg_ cells support liver fibrosis by influencing metalloproteinases activation (Zhang X. et al., 2016). T_H_17 cells further signal on non-lymphoid cells such as endothelial cells, fibroblasts, and keratinocytes inducing the production of inflammatory cytokines such as IL-6, GM-CSF, IL-1, TGF-β, TNF-α, and MCP-1 to attract immune cells thereby promoting pro-inflammatory immune responses. IL-17 receptor signaling further activates the expression of antimicrobial peptides and matrix metalloproteinases, whereas the latter are important for the degradation of scar tissue during infection (Bettelli et al., 2008). In patients with more severe liver cirrhosis, an increased frequency of circulating T_Reg_ cells, but a decreased T_Reg_**/**T_H_17 ratio was positively correlated with disease progression (Li et al., 2012). More particularly, the abundant expression of TGF-β and IL-6 in the liver, which both favor the differentiation of T_H_17 cells, points to a major contribution of these cells during this process. Liver cirrhosis is one of the major risk factors for the development of HCC. Thus, 80%–90% of HCC develop in the course of chronic inflammation (Refolo et al., 2020). HCC is associated with a poor prognosis, is hard to detect, is aggressive, and has limited therapeutic options. T_Reg_ and T_H_17 cells were increased in tumor tissue in comparison to the surrounding liver tissue. Not only high intra-tumoral T_Reg_ frequencies and a decreased T_H_17 quantity but also CD39 expressed by tumor cells and T_Reg_ cells facilitated HCC growth, metastasis, and poor prognosis by mainly affecting T_H_17 function and differentiation (Bettini et al., 2012). T_Reg_ cell depletion, however, negatively influenced HCC growth (Cany et al., 2011). Interestingly, high IL-17 and IL-17R expression in the tumor tissue and elevated circulating T_H_17 cells are also associated with poor survival and early HCC recurrence. Sorafenib is currently used in HCC treatment and targets varies kinases expressed in T_Reg_ cells. This therapy negatively affects the T_Reg_ frequency, which can be correlated to overall improved survival (Voron et al., 2014). To improve the overall survival of HCC patients, new therapeutic approaches are essential. A defect in T cell function is described and immunotherapies are potential effective treatment strategies. One possibility could be checkpoint inhibition. PD-1 upregulation for example can be detected in circulating T_Reg_ cells in HCC patients, supporting the overall immune dysregulation. Therefore, anti PD-1 is a promising strategy. This therapy reverses the T_Reg_ mediated inhibition of T_H_17 cells and other effector T cells (Langhans et al., 2010) (**Figure 4**).

### Liver Transplantation

Liver transplantation is in many cases the only possibility to cure end-stage liver disease. The short-term outcome has significantly improved, but chronic organ rejection and the side effects of immunosuppressive therapy remain a concern. The optimal immunosuppressive treatment to prevent organ rejection and toxicity and at the same time avoid opportunistic infections must be tightly balanced and will vary between individuals. The state of optimal immunotherapy, called operational tolerance, is difficult to achieve, and most patients require a life-long therapy with numerous side effects. Thus, new therapeutic approaches following liver transplantation are urgently needed.

T_Reg_ cells play a leading role in averting the cause of graft-versus-host disease (GvHD) which leads to organ rejection. T_Reg_/T_H_17 balance plays a fundamental role in rejection pathogenesis (Wang et al., 2019), and T_H_17 cells were found to be elevated during acute and chronic organ rejection. In addition, the pro-inflammatory cytokine milieu during organ rejection can induce RORγt and IL-17 expression in T_Reg_ cells. Thus, T_Reg_ cells seem to contribute to organ rejection. Indeed, an early decrease in T_Reg_ frequency is a risk factor for suspected acute and biopsy proven acute rejection (Han et al., 2020). In addition, CD39 expression in the transplanted liver tissue influences the T_Reg_ and T_H_17 immune response and thereby organ rejection and GvHD (Yoshida et al., 2015). Adoptive T_Reg_ transfer was protective in mice, and currently several clinical trials are testing the efficiency and safety in humans (Romano et al., 2017). Another study tested the T_Reg_ therapy in patients 6–12 months after liver transplantation and demonstrated an increase in circulating T_Reg_ cells and reduced anti-donor T cell response (Sanchez-Fueyo et al., 2020). Although the long-term effects are not evaluated, T_Reg_ therapy could be a promising therapeutic approach. In contrast to the beneficial effects, in transplant patients with HCV infection, early high levels of T_Reg_ cells and T_H_1 cells after liver transplantation are associated with severe recurrent HCV infection (Ghazal et al., 2019).

In addition to T cell-mediated organ rejection, danger associated molecular patterns (DAMPs) play an important role in triggering sterile inflammation in the liver after organ transplantation. Sterile inflammation could be detected in different solid organs after implantation, and sterile inflammation influences the transplant tolerance and chronic rejection. A recently published study described the correlation between elevated DAMPs and acute postoperative multi-organ dysfunction (Nagakawa et al., 2020). In sum, several studies implicate the contribution of sterile inflammation in acute and chronic organ rejection and are reviewed elsewhere (Braza et al., 2016) (**Figure 4**).

## Conclusion

T_H_17 and T_Reg_ cells are essential in orchestrating the intrahepatic immune response in health and disease. Under homeostatic conditions their balance must be tightly regulated to have an effective immune response and to prevent tissue damage. In the course of several diseases, their balance is shifted. Especially in the liver, the T_H_17/T_Reg_ response is tremendously important. An overwhelming T_H_17 response, for example in NASH, can promote the inflammatory state of disease and is associated with disease progression. On the other hand, T_Reg_ cells prevent the anti-tumoral immune response in HCC and promote metastasis and cancer growth. Furthermore, both cell subsets can be beneficial in different liver diseases settings. Thus, T_H_17 cells are necessary for effective pathogen clearance in the liver and T_Reg_ cells are important to coordinate the immune response in autoimmunity and after liver transplantation. Most of the findings on the role of T_H_17 and T_Reg_ cells were generated in mice. The importance of investigating their influence in humans is highlighted in several disease settings. Many studies demonstrate the necessity of investigating the function and quantity of these cells in liver tissue specifically, as circulating cells may not reflect intrahepatic conditions. In addition to altered function and quantities in liver diseases, T_H_17/T_Reg_ cell balance could be a therapeutic target in disease settings.

Future studies should investigate the function of T_H_17 and T_Reg_ cells in liver tissue and review their balance. Furthermore, different signals that might influence T_H_17**/**T_Reg_ cells, especially in the liver, must be analyzed to fully understand the liver-specific pathomechanism and to inform a liver-specific therapy.

## Author Contributions

Conceptualization: HD and LB. Writing—original draft preparation: HD, LB, and RW. Visualization: SW. funding acquisition: HD, LB, and RW. All authors contributed to the article and approved the submitted version.

## Funding

The research was funded by the German Research Foundation (RW: SFB/TRR57, HD: DR 1161/1-1 LB: BA 7175/1-1).

## Conflict of Interest

The authors declare that the research was conducted in the absence of any commercial or financial relationships that could be construed as a potential conflict of interest.
